# Book Review: Social Research Methods: Qualitative and Quantitative Approaches

**DOI:** 10.3389/fpsyg.2021.696828

**Published:** 2021-05-25

**Authors:** Irene Khosla

**Affiliations:** Discipline of Psychology, School of Social Sciences, Indira Gandhi National Open University, New Delhi, India

**Keywords:** research, qualitative research, quantitative research, review, social sciences

## “The Art and Science of Asking Questions is the Source of All Knowledge”—Thomas Berger

In an endeavor to bridge the gap between knowledge and applicability, Neuman (2014) presents a meticulous and comprehensive amalgamation of concepts and theories, defining qualitative and quantitative research methods in his book “Social Research Methods: Qualitative and Quantitative Approaches.” A professor of sociology at the University of Wisconsin-Whitewater, William Lawrence Neuman has gained immense experience and has worked rigorously in his subject matter. He has authored seven books, numerous book chapters, and articles in the field of social sciences.

The book reviewed at present is the seventh edition of the “Social research methods: Qualitative and Quantitative Approaches,” which was published by Pearson Education Limited in 2014. The book was written to help aspiring researchers gain an in-depth understanding of research and its purpose while stressing the essentials and theoretical considerations of conducting research. With a total of 15 chapters, the book elucidates various research methods, balancing between qualitative and quantitative approaches with an aim to emphasize the conceptual framework, applications, strategies, and the pros and cons of each approach, along with highlighting the benefits of using a combination of the two approaches.

The current edition is divided into five parts—foundations of research; planning and preparation for research; quantitative research methods—collection and analysis of data; qualitative research—methods of collecting data and analysis; and lastly “communicating the results of research with others.”

Part one of the book consists of five chapters, shedding light on the basics to provide an understanding of the and how of research and its importance; types of research; theoretical conceptualizations; methodology; and conducting a literature review and ethics in research. For example, in chapter one, the author explains the need to learn how to conduct research, followed by explaining the use, scope, and target audience for research in chapter two. Furthermore, in chapter four, in the most beautifully structured manner, the author has elaborated on the philosophical foundations and paradigms of research.

Part two describes the basics of the process of conducting research. Divided into three chapters explaining qualitative and quantitative research in terms of—research designs and its various strategies; measurement of data; and sampling. Research design issues, reliability and validity, and the types of scales and inventories used are also discussed in this section, providing an integrative and inclusive view of the research process.

In the third part, the types of research and their processes are elaborated for collection of data and analysis in quantitative research. Spread across four chapters, the topics covered under this section include experimental research; survey research; non-reactive research and secondary analysis; and quantitative analysis of data. Whereas, the fourth part is dedicated to qualitative research. Described in two chapters, this section focuses on field and focus group research and analyzing qualitative data.

Parts three and four of the book do justice to the concepts by providing thorough information about the procedure and methods of research. It covers the history, advantages, disadvantages, uses, requirements, as well as gives details about the types of variables and statistical and non-statistical techniques that can be applied. Each chapter is enriched with figures, diagrams, and maps which aid in enhancing conceptual clarity. For example, chapter ten includes information about the latest technological advances such as online surveys and computer-assisted data collection and chapter 14 includes detailed figures depicting qualitative data analysis techniques, with a figure for each like narrative analysis.

The last part of the book and the final chapter provides detailed information on writing and publishing research reports as well as talks about the politics in social research. This part covers everything from why a research report is required, to understanding the writing process, formulating a research proposal, to discussing the ethics, limitations, advantages, and difficulties faced in conducting and publishing research.

In terms of the structure of the book, each chapter begins with the title and key pointers of the topics to be discussed, along with a quote or a small paragraph, which in a theoretical yet poetic style serves as a brief introduction to the topic. Needless to say, each topic mentioned is covered scrupulously and thoroughly in a holistic manner and is explained in-depth, clearly divided point-wise and under sub-categories. This helps in reducing the burden of information overload and aids in maintaining the readers interest.

The most noteworthy and distinguishing part of the book is the use of alternate means of representing and expressing information. Each topic is supported with various realistic examples, enriched with numerous figures, maps, diagrams, and is summarized in organized and structured tables for comparison and ease of understanding. The author has also included dialogue boxes in each chapter with short definitions of the topics in discussion. This is advantageous from a learning perspective as it provides a quick glimpse, simplifying the comprehension of concepts. It is these features that give the book an edge over other books of research.

The book also incorporates empirical evidence and statistical data in supporting its content and illustrations, making it more credible. The language used is simple and straightforward yet catchy in terms of grasping the reader's attention, making even complex theories and perspectives intelligible. At the end of each chapter, a list of key terms is provided, followed by a set of review questions. These questions are beneficial as a means of assessing conceptual clarity in addition to encouraging the reader to ruminate and indulge in lateral thinking over the subject matter.

Overall, the book is a valuable asset for the field of research. The confluence of theoretical concepts with realistic examples makes the book highly applicable and significant not just for students, but for anyone keen to venture into the realm of social research. Just like a building cannot withstand without a strong foundation, a researcher cannot exist without building and maintaining their repositories of knowledge. In conclusion, the book is a quintessential means of grasping and gaining mastery over research knowledge.

## Author Contributions

The author confirms being the sole contributor of this work and has approved it for publication.

## Conflict of Interest

The author declares that the research was conducted in the absence of any commercial or financial relationships that could be construed as a potential conflict of interest.
